# Computer-aided analysis of micro-morphological structure of porous membranes

**DOI:** 10.1186/s12938-018-0481-9

**Published:** 2018-05-30

**Authors:** Juliusz L. Kulikowski, Malgorzata Przytulska, Andrzej Chwojnowski

**Affiliations:** Nalecz Institute of Biocybernetics and Biomedical Engineering PAS, Warsaw, Poland

**Keywords:** Porous membranes, Porosity evaluation, Image processing, Membrane’s quality assessment

## Abstract

**Background:**

The paper presents an approach and computer-aided method of numerical evaluation of the quality of porous membranes used as scaffolds for cultivation of chondrocytes in regeneration of biological tissues.

**Materials:**

The scanning electron microscope (SEM) images of 300× and 1000× magnification presenting the sections of artificial polyvinylpyrrolidone membranes obtained in two alternative production processes are examined.

**Theory and methods:**

There is presented a combined morphological and statistical method of the assessment of artificial membranes’ porosity, based on computer-aided segmentation and analysis of the size and shape of pores. Theoretical backgrounds of description pores as irregular objects in discrete 3-dimensional space are presented. The parameters characterizing the quality of pores: pores irregularity coefficient and pores density are defined. The quality of the examined specimens of materials is characterized by the size (mean 2-dimensional section areas) of pores. The main concept presented in the paper is the extraction of lacking information concerning the third dimension of pores from the 2-dimensional SEM images of their sections. Two approaches to evaluation of the parameters characterizing pores on the basis of computer-aided analysis of their cross-sections are proposed: (1) based on statistical extension of geometrical data and (2) based on analysis of brightness profiles. The corresponding methods are based on the assumption of isotropy of the examined porous materials. The results of automatic measurements of the areas of pores, lengths of their chords and recording the brightness profiles along fixed lines crossing the analyzed images are illustrated by examples.

**Conclusions:**

Practical usefulness of the proposed methods to evaluation of the quality of porous membranes consists in their ability to be used in case if alternative methods for some reasons cannot be used.

## Background

Numerous physiological processes taking place in living organisms: absorption of nutritive products and gases, excretion of waste products etc. are based on the phenomenon of diffusion of various types of material particles through porous membranes. Similar processes play a substantial role in numerous areas of medical therapy based, in particular, on artificial organs: artificial lungs-heart, artificial kidney, encapsulated drugs or living cells, cultivation of artificial tissues etc. [1–5]. Porous membranes application to water or air purification indirectly influences also human health [6–8]. In all the above-mentioned cases porous membranes play the role of filters separating some biochemical media or of scaffolds for seeding and cultivation of tissues. The mechanisms of separation or of settling particles (cells) depend on biophysical, mechanical and micro-structural properties of the porous materials. Examples of biophysical properties are electric- and heat-conductivity, dielectric constant, pH level, etc. Mechanical properties denote specific gravity, Young module, break- or fracture-resistance, etc. The notion of micro-structural properties concerns spatial distribution, density, size and shape of the components the investigated material consists of, in particular—of pores and/or of the walls that separate them.

**Fig. 1:**
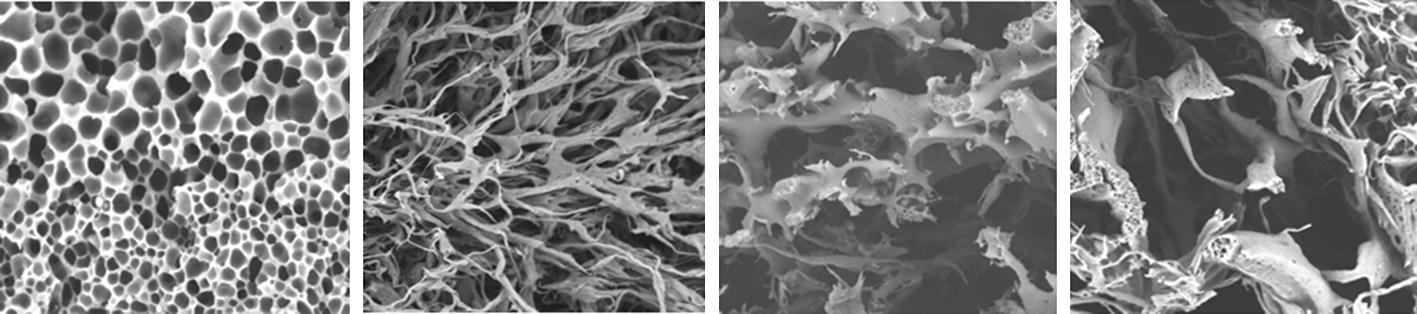
Examples of electron microscope images of various porous materials’ cross-sections

It was shown in several former papers [9–13] that computer technology combined with adequately chosen microscopic imaging methods may be an effective tool for material’s porosity analysis and evaluation. The 2D cross-sections of the samples of porous materials provide interesting but highly intricate images of magnified pores as cavities and channels penetrating the material, as shown in Fig. 1.

In most cases the pores are of different size and highly irregular shape. However, the notion of *irregularity* needs some explanation. We call *regular* curves (contours) or 3D shapes which can be precisely described by simple analytical functions or geometrical objects. *Weakly irregular* are contours or 3D shapes which can be described as parameterized compositions of finite number of regular curves (contours) or 3D shapes. Curves (contours) or 3D shapes are *strongly irregular* if they are neither regular nor weakly irregular. The class of strongly irregular curves (contours) and 3D shapes can be divided into the sub-classes of *random* and *chaotic* objects. *Random* curves (contours) or 3D shapes are assumed to be able to be described by probabilistic models [14, 15]. Chaotic objects can be described by *fractals* [16] or any other, more sophisticated, theoretical tools. Below, it is assumed that pores with sufficient accuracy can be described as random-shape objects. Therefore, no geometrical model is suitable for their accurate description. The statistical geometry or morphology based methods [17, 18] seem to be more suitable for numerical analysis of this type of structures. However, considering images of pores as instances of specific 2D random fields seems to be more rational. On the other hand, porosity is a 3D rather than a 2D property of a rigid body. Therefore, it arises a non-trivial problem of obtaining information about the 3D structure of a porous material by analysis of images presenting its 2D cross-sections. The problem is relatively simple if the samples of the investigated material can be cut into regularly distanced parallel slices, as proposed in [12, 19] Unfortunately, this cannot be done, e.g. in the case of high fragility of the samples. In such case, we are faced with a more sophisticated statistical decision making problem to be solved by using advanced computer-based methods. In this paper some concepts concerning overcoming the difficulty of 3D morphological structures description not based on analysis of stacks, but of single 2D images, are presented. The missing information about the 3rd dimension of the examined porous materials’ samples can (at least partially) be extracted from the brightness level distribution in the cross-section images or should be deduced under the assumption of 3-dimensional morphological isotropy of the samples. Below, both methods will be shortly described. However, statistical porosity characteristics describe only some aspects of the investigated materials’ utility in their biomedical applications. The relationships between measurable morphological parameters and large-scale properties of porous materials are still an open problem requiring additional observations and experiments and it is not considered here.

The analysis of irregular structures (in particular, of pores) is not a new problem. Its origins date the Buffon’s needle problem which initiated the development of stochastic geometry [20]. Roughly speaking, this area of investigations concerns the properties of random compositions of regular geometrical objects. This is not quite the problem of highly irregular shapes investigation arising in biomedical engineering, material engineering, geophysics, geomorphology, etc. [11, 14, 17, 21]. However, each of the disciplines mentioned here uses its specific experimental data acquisition methods and this causes that the corresponding data processing methods are not directly applicable in other application areas. For example, echo-sounding methods of geophysical data acquisition and SEM imaging of pores provide formally different types of experimental data, despite the fact that in both cases they concern less or more irregular morphological structures.

The paper is organized as follows. The “Materials” section shortly presents the materials used as a source of image data subjected to computer analysis. The “Theory and methods” section consists of four subsections. The “Preliminary image processing” subsection presents initial operations aimed at enhancement the quality of SEM images before their analysis. The “Basic assumptions” subsection describes some discrete geometry objects used in computer analysis of pores. The “Characteristics of irregular object” subsection contains definitions of basic parameters used to describe irregular shapes of pores. The “Rough evaluation of 3D objects’ porosity” subsection is devoted to presentation of proposed two approaches to evaluation of the volumes of pores on the basis of analysis of their 2D sections: 1st based on statistical extension of geometrical data and 2nd based on analysis of brightness profiles. “Conclusions” contain remarks summarizing the work.

## Materials

The materials used in this study have been provided by the Laboratory of Semi-permeable Membranes and Bioreactors of the Nalecz Institute of Biocybernetics and Biomedical Engineering, Polish Academy of Sciences. They had the form of series of images presenting the cross-sections of samples of porous membranes produced in the Laboratory. The membranes were produced by an inverse-phase method using the Poli-l-lactide (PLLA), and polyvinylpyrrolidone (PVP) as basic input components [22]. Several variants of technological processes were used in order to select the most effective ones considering the highest membrane’s quality and the highest production process stability. The samples of investigated materials were cut and covered by a 7‒10 nm coat of gold and in this form they have been used as specimens for analysis in a scanning electron microscope (SEM) type Hitachi TM1000 using a 15 kV acceleration voltage. The cross-section images in 300× and/or 1000× magnifications in electronic form of (approximately) 1100 × 1280 pix TIFF standard visual objects were then provided for computer analysis.

## Theory and methods

### Preliminary image processing

All calculated morphological characteristics of pores are based on the distances expressed in pixels (pix), areas expressed in pix^2^ etc., recalculated to metric units. In order to make the parameters evaluated in various samples of material mutually comparable it is necessary to present the images in standardized size, magnification and scale (in pix/μm). Moreover, in order to outline the pores more exactly the contrast of the original SEM images should be enhanced. Typical image enhancement methods are described in [23, 24].

**Fig. 2:**
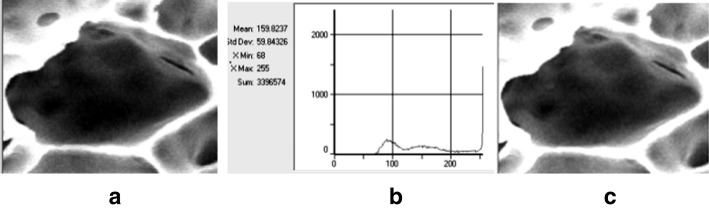
A typical cross-section of a pore: **a** original image, **b** its brightness histogram, **c** image enhanced by its histogram extension to the left side of brightness scale

Figure 2 shows a typical cross-section of a pore: original SEM image (a), its brightness histogram (b) and the image enhanced by its histogram extension to the left side of brightness scale (c). This operation makes the borders and inner details of pores better visible, however, it changes the original relationship between the brightness level and the depth of the visible pores insight.

### Basic assumptions

**Fig. 3:**
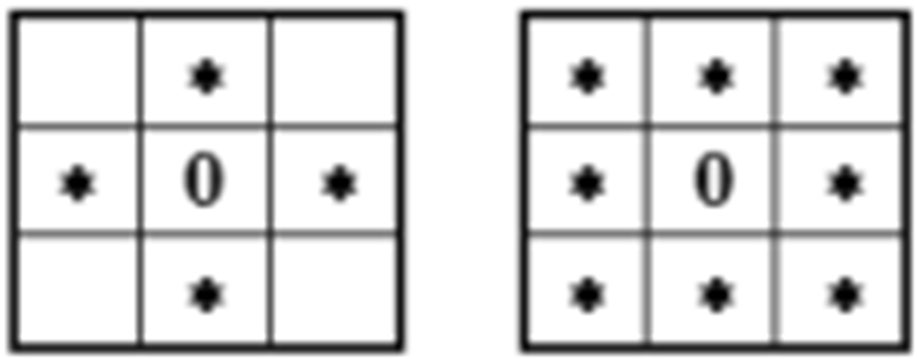
Schemes of connectivity of points in a 2D discrete space

Morphological analysis of pores is aimed at investigation of their size, shapes and spatial distribution. As such, it is based on some topological and metrical notions. Original SEM images can be considered as objects imbedded in Euclidean (2- or 3-dimensional) space, while their representatives subjected to computer analysis are objects of discrete (2D or 3D) spaces. The difference between the two types of formal models is particularly substantial if small-size objects are taken into consideration. This is caused by the fact that distances between any pair of points in Euclidean space can be expressed by any non-negative real number while in discrete space defined on a rectangular grid of points they are limited to enumerable sets of values, depending on an assumed type of connectivity of adjacent points. Pairs of different points can be called connective when their absolute (Manhattan) distances *d*: (a) satisfy the condition *d *= 1 or (b) satisfy the condition *d *≤* n* where *n* is the dimensionality of the space. As a consequence, in 2D space two types of connectivity: 4-connectivity and 8-connectivity can be established, as it follows from the below shown tables of points (⋇) adjacent to a central point (0) (Fig. 3).

**Fig. 4:**
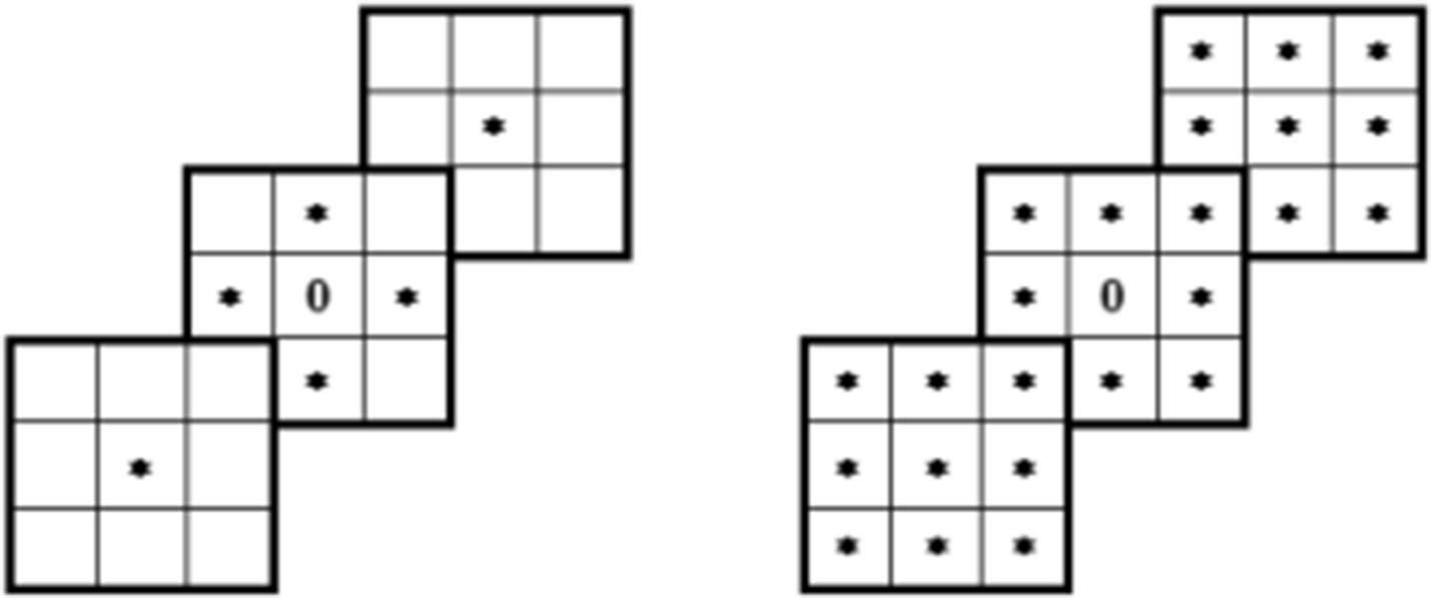
Schemes of connectivity of points in a 3D discrete space

Similarly, in a 3D space a 6-connectivity and 26-connectivity can be established, as illustrated by the tables (Fig. 4).

An *open discrete line*-*segment* can be defined as a minimal linearly ordered set of points *Λ *= [*p*_*0*_, *p*_*1*_…, *p*_*κ*_,…, *p*_*K*_] such that *p*_*0*_ and *p*_*K*_ are connective to exactly one, while all the others to exactly two other points of the set. A *closed discrete line segment* (a *contour*) *C* is defined similarly excepting that it does not contain any point connective to less than two other points of the segment (*p*_*K*_ is connective both to *p*_*K−1*_ and *p*_*0*_).

A *finite discrete 2D* or *3D object* can be defined as a finite set *Ω* = {*p*_*κ*_}, *κ* = 1,2,…,*K*, such that each point *p*_*k*_ is connective to at least one other point of *Ω*. The *surface* of *Ω* is defined as a maximal subset *S*_*Ω*_⊆ *Ω* such that each its point *p*_*κ*_ is connective to at least one point not belonging to *Ω*. An object *Ω* is called *thin* if *S*_*Ω*_≡ *Ω*. Evidently, contours and surfaces of objects are thin objects themselves. The above-defined discrete objects have also a property of compactness, i.e. any pair of their points can be linked by a line totally belonging to the object.

A *discrete length* of a line segment *Λ* or of a contour *C*, measured in pixels [*pix*], is defined as the number of points constituting them. Similarly, a *discrete area* of a surface *S*_*Ω*_, measured in [*pix*^2^] and *a discrete volume* of an object, measured in [*pix*^3^], are defined as numbers of points that constitute them.

**Fig. 5:**
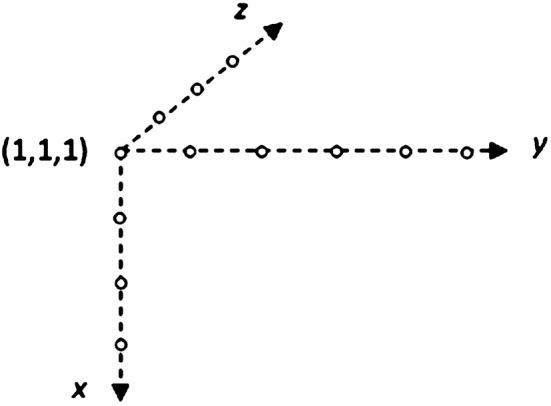
System of discrete coordinates used for description of irregular objects

The discrete 3D objects will be described in a left-handed system of coordinates (*x*, *y*, *z*), adequate to be presented on a computer screen, as shown in Fig. 5.

The higher-connective models suit, in general, better to approximation of continuous shapes then the low-connective models. However, evaluation of distance, area or volume measures in discrete models of geometrical objects by counting the numbers of pixels belonging to the objects may lead to substantial differences concerning the respective Euclidean measures of the objects. This should be taken into account in morphological analysis of pores.

### Characteristics of irregular objects

The above-defined geometrical notions will be used to describe some basic properties of pores. In particular, the distance and/or area measures can be used as basic data to evaluate morphological characteristics of irregular shapes of natural objects. A 2D contour of a pore’s cross-sectio*n* can be roughly characterized by its *length L*, *area S* and *regularity* coefficient:

1 $$\eta = 4\pi \frac{S}{{L^{2} }}$$

taking value * η*=1 in circles and decreasing to 0 if the shape of contour becomes more irregular.

Similarly, a *regularity* coefficient of a 3D object can be defined as:

2 $$\gamma = \kappa \frac{V}{{\sqrt {S^{3} } }}$$

where *V* denotes the volume of the object,

2a $$\kappa = \frac{{3\sqrt {\pi^{3} } }}{4\pi } \cong 1,329$$

and *γ *= 1 in spheres and it is decreasing to 0 if the shape of a 3D object (pore) becomes more irregular.

**Fig. 6:**
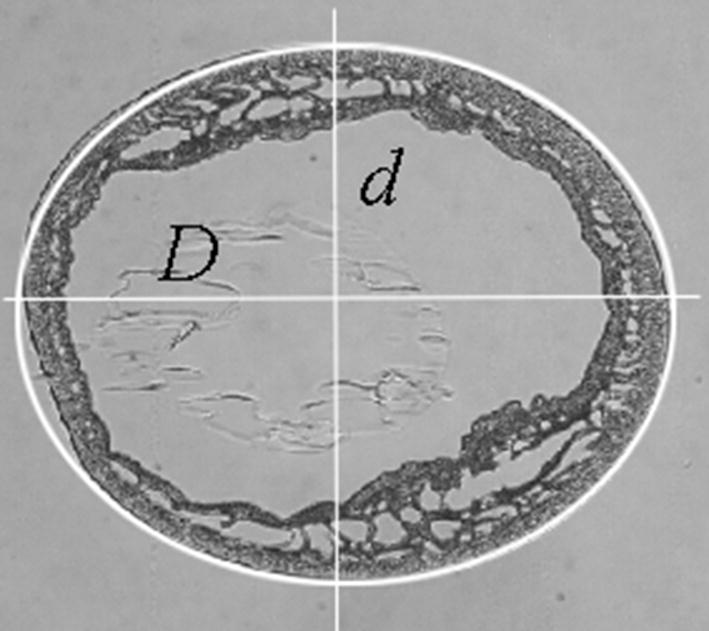
Example of a microcapsule outlined by ellipse

The above-given formulae suit well if the input data *L, S* and/or *V* can be exactly measured. This takes place, if a 2D object has been outlined, as shown in Fig. 6 where a cross-section of a microcapsule has been outlined by an ellipse.

If the larger *D* and smaller *d* diameters of the ellipse are parallel to the screen axes (as it, approximately, takes place in Fig. 6) then the Euclidean lengths of the diameters are given by the formulae:

3a $$D_{E} = \delta \cdot D,$$

3b $$d_{E} = \delta \cdot d$$

where *D* and *d* are (both in case of 4- and 8-connectivity) expressed by the number of pixels and *δ* denotes a scale factor given in metric units (e.g. in μm) per pixel. Then the well-known formulae for ellipses can be used:

4 $$L \cong 2\pi \cdot D \cdot \left( {\frac{3}{4} + \frac{{d^{2} }}{{4D^{2} }}} \right),$$

5 $$S = \pi \cdot d \cdot D.$$

and, from (1),

6 $$\eta = 4 \cdot \sqrt {\frac{d}{D}} \cdot \frac{{D^{2} }}{{3D^{2} + d^{2} }}$$

However, the situation differs in a more general case when the distances and areas in a discrete space 2D (both, in the 4- and 8-connectivity case) are expressed by the numbers of pixels constituting the objects (lines or contours’ inside). In such case the numerical values of contour lengths or of areas not only differ from those in Euclidean space but also depend on the type of connectivity and on the size and spatial orientation of the objects. This is show in Table 1 where four circles of different radii have been approximated by their discrete representatives and their regularity coefficients *η* have been calculated.

**Table 1:** Regularity coefficients *η* calculated in various ways

Type of connectivity/radius	4-connectivity			8-connectivity			Continuous (Euclidean)		
	*L*	*S*	*η*	*L*	*S*	*η*	*L*	*S*	*η*
1	8	9	*1.767*	4	5	*3.927*	6.283	3.142	*1*
3	24	45	*0.982*	16	37	*1.816*	18.850	28.274	*1*
5	40	97	*0.762*	28	96	*1.539*	31.416	78.540	*1*
10	80	349	*0.685*	56	348	*1.394*	62.832	314.159	*1*

The differences between the regularity coefficients are given in italics

It follows from the Table that assumption of 8-connectivity of the discrete space suits to describe irregular contours better than 4-connectivity. Moreover, in both cases the regularity coefficient *η* is better suited for characterization of larger than of small objects. In any case, the type of connectivity should be a priori strongly established in order to make the results of porosity evaluation mutually comparable. However, it still remains the problem, how to evaluate the values of *S* and *V* of 3D objects observable only in 2D space without a possibility to measure them directly in all three dimensions.

### Rough evaluation of 3D objects’ porosity

The differences between the discrete and continuous measures of distance, area, or volume may be less substantial if morphological parameters based on them are used only to comparative analysis of porous materials. In such case the easier measurable discrete (8-connective) values *L*_*i*_ and *S*_*i*_ of outlined pores (*i* enumerating the pores in the image) within the frame of an examined SEM image can be evaluated. For them the regularity coefficients *η* can be calculated. The following parameters are widely used as basic porosity characteristics [13, 25]:

- Mean pores’ volume *m*_*V*_,
- Standard deviation of pores’ volume *∆*_*V*_,
- Total pores’ density:
  7 $$\rho = \frac{{V_{\sum } }}{V}$$

where *V*_*Σ*_ denotes a total volume of pores in the volume *V* of the analyzed specimen. The density *ρ* may vary between 0 and 1.

In order to calculate the above-listed parameters the volumes *V*_*i*_ of pores in the observation area should be evaluated. In [26] a method of statistical evaluation of *V*_*i*_ based on the assumption of isotropy of the morphological structure of pores and of their quasi-spherical shapes was described. Below, two other approaches to solution of the problem are presented.

#### Approach based on statistical extension of geometrical data

**Fig. 7:**
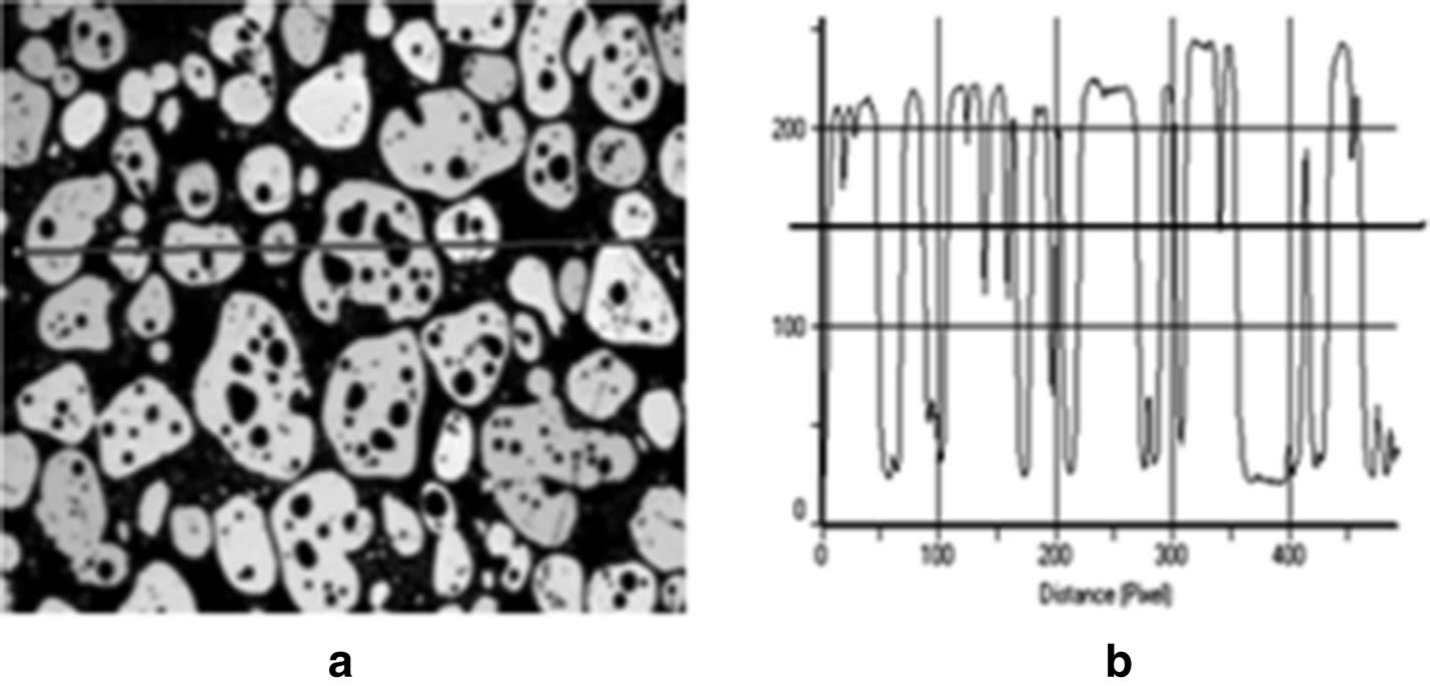
Brightness profile of an anisotropic porous structure: **a** cross-section perpendicular to the axes of fibers, **b** its brightness profile along a horizontal line crossing image **a**

This approach is particularly suitable to analysis of pores whose shapes can roughly be predicted by taking into consideration the method of porous material production. In particular, porosity can be caused by leaving free areas in a squeezed bunch of fibers. In such case the structure of pores is anisotropic: irregular (with holes) in a plane perpendicular to the dominating direction of fibers and lengthened along the axes of fibers. Example of such porous structure is shown in Fig. 7.

In Fig. 7a brightness values varying mostly near two extreme levels: the highest level corresponding to the cross-sections of fibers and the lowest, dark level corresponding to the pores are remarkable. This suggests that the pores density *ρ* in this case can be given approximately by the formula

8 $$\rho \cong \frac{{S - S_{\sum } }}{S}$$

where *S* denotes the area of the image frame and *S*_*Σ*_ stands for total area of all white spots in the frame corresponding to the cross-sections of the fibers.

Another situation arises in the case of isotropic type of porosity. Evaluation of porosity can then be based on the following assumptions.

##### **Assumption 1**

The morphological structure of the examined porous material is isotropic in 3D; therefore, the probability distributions of the lengths of parallel chords crossing the pores do not depend on the direction of the chords.

##### **Assumption 2**

The cross-sections of the pores in (*y*, *z*) plane are of elliptic form with the major and minor axes ordered in parallel to the *y* and *z* coordinate axes.

##### **Assumption 3**

The pore’s cross-sections in the (*x*, *y*) plane are of compact irregular form.

Assumption 3 seems to suit better to characterization of irregular shapes of pores than this based on the concept of effective pore diameter proposed in [27–29].

**Fig. 8:**
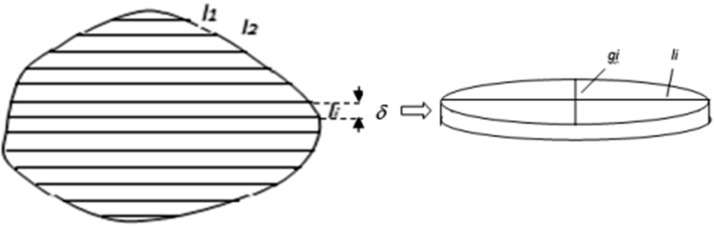
Measuring parallel chords in a pore’s contour

Figure 8 presents a typical contour of a pore, as visible in (*x*, *y*) plane, divided into horizontal strips of equal breadths *δ*.

**Fig. 9:**
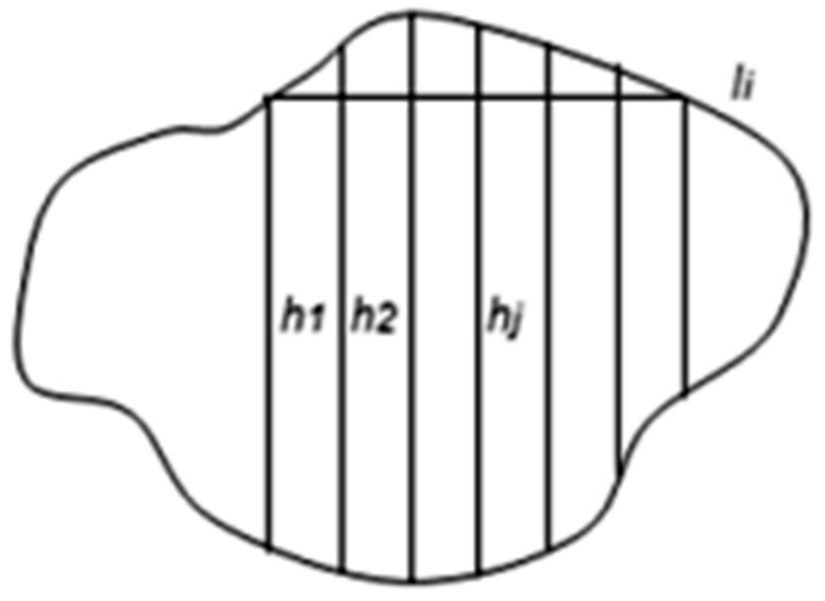
Vertical chords assigned to a fixed horizontal chord of a contour

The lengths of so-obtained horizontal chords are denoted by *l*_*i*_. According to Assumption 2, the partition of the contour into strips corresponds to cutting the 3D pores into horizontal disks of a constant thickness. The disks then can be approximated by flat cylinders of elliptic base and constant height. The chord length *l*_*i*_ can be taken as an axis of the elliptic base of the cylinder, the other (perpendicular) axis should be directed along the (invisible) coordinate *z*. In order to calculate the volume of the cylinder:

9 $$\nu_{i} = \frac{\pi }{4} \cdot \delta \cdot l_{i} \cdot g_{i}$$

the length *g*_*i*_ of the second axis should be evaluated. For this purpose Assumption 1 justifying the proposed statistical extension approach will be used. For any given, *i*-th horizontal chord a set of crossing it vertical equally distanced parallel chords of the pore’s contour should be found and their lengths *h*_*1*_, *h*_*2*_,…, *h*_*J*_ should be measured, as shown in Fig. 9. For the sake of simplicity, the distances between the vertical chords can also be fixed equal *ε*, *ε* > 0, in particular, *ε* = *δ*.

Thus, it is assumed that for any given (*i*-th) horizontal chord a conditional probability distribution (*cpd*) of the length of the assigned to it vertical chords *w*(*h*|*l*_*i*_) can be taken into consideration and the same *cpd* is in effect also for the length *z* of horizontal chords crossing the *i*-th chord and directed perpendicularly to the visualization plane.

Let us consider the set *Φ*_*i*_ = {*h*_*1*_, *h*_*2*_,*…*,*h*_*J*_} of measured lengths of the vertical chords. Two approaches to evaluate the missing value *g*_*i*_ on the basis of *Φ*_*I*_ can be taken into account:

- Based on the mean value:
  10 $$g_{i} = M_{i} = \frac{1}{J}\sum\limits_{j = 1}^{J} {h_{j} } ;$$
- Based on the highest conditional probability.

This approach is reasonable only in case of large values of *J* (e.g. *J *≥ 10). For this purpose let us find the minimal and maximal *h*_*max*_ values *h*_*min*_, *h*_*max*_ ∈ *Φ*_*i*_. Then, for a chosen value *K*, 0 < *K *<* J* the length of an auxiliary sub-interval:

11 $$c = \frac{{h_{\hbox{max} } - h_{\hbox{min} } }}{K}$$

should be calculated. For *k*, 1 ≤ *k* ≤ *K*, the numbers *n*_*κ*_ of *h*_*j*_ ∈*Φ* satisfying the inequalities:

12 $$(\kappa {-} 1) \cdot c < h_{j} \le k \cdot c,\quad \kappa = 1, 2, \ldots ,K.$$

are then defined and should be calculated.

Let κ* be such that *n*_κ*_ = *max*[*n*_*1*_, *n*_*2*_,…, *n*_*K*_]. If *n*_κ*_ is unique then we put:

13 $$g_{i} = \left( {\kappa * - \frac{1}{2}} \right) \cdot c.$$

Otherwise, if more than one sub-interval satisfies the condition of maximization *n*_*κ*_ the number *K* should be reduced by 1 and the procedure should be repeated. In a marginal case, when *K* = 1 is reached, all *h*_*j*_ have approximately the same value h* and *g*_*i*_ = h* as a solution can be accepted.

Finally, the values *g*_*i*_ given by (10) or (13) can be used in formula (9) for calculation of the volumes *v*_*i*_ and

14 $$V_{\sum } \cong \sum\limits_{i = 1}^{I} {v_{i} }$$

can be used in formula (7) for calculation of the pores density *ρ*.

#### Approach based on analysis of brightness profiles

**Fig. 10:**
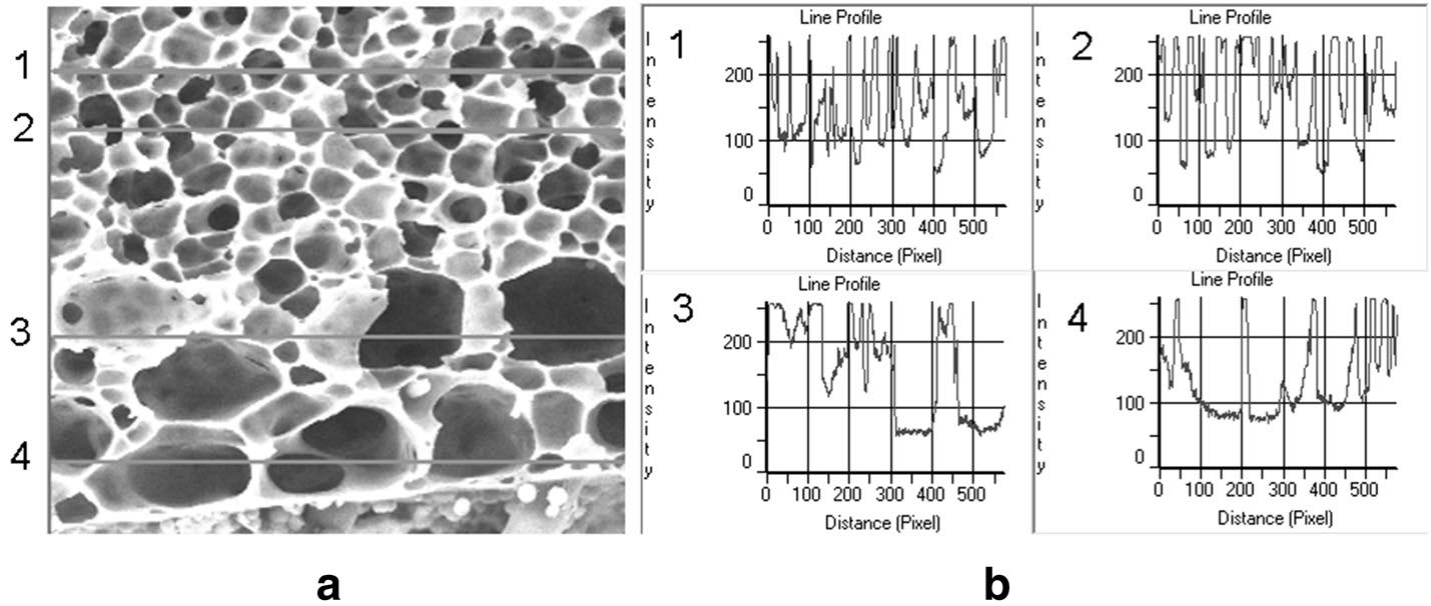
Pores cross-section: **a** SEM image with plotted horizontal lines fixing the brightness profiles, **b** four corresponding brightness profiles

In this section porous materials whose pores have inner shape similar to this shown in Fig. 10a are considered.

The visualized pores are not only of various sizes and shapes but also of various brightness caused by their different depths and inner profiles. The profiles recorded along several horizontal lines crossing the image, shown in Fig. 10b, 1–4, suggest a possibility of extraction of information concerning the depths of pores in the third dimension. Therefore, the below presented method will be based on the following:

##### **Assumption 4**

The brightness levels in 2D visualization of pores are linearly proportional to the metric depths of pores with negative coefficient of proportionality.

**Fig. 11:**
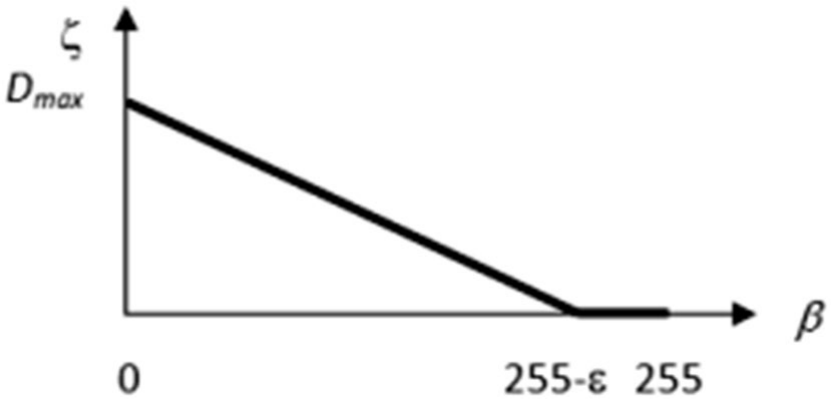
Assumed relation between brightness level *β* and assigned to it depth of pores ζ

Moreover, the former Assumptions 1 and 3 hold as well. Taking into account that the recorded brightness levels *β* are given by the integers satisfying the inequality 0 ≤ *β *≤ 255, the following relation between *β* and the corresponding depth ζ of a pore can be established:

15 $$\zeta = {\text{ }}\left\{ {\begin{array}{*{20}l} {D_{{max}} (1 - q \cdot \beta ){\text{ }}\quad {\text{for}}\quad 0 \le \beta < 255 - \varepsilon ,} \\ {0\quad {\text{for}}\quad 255 - \varepsilon \le 255,} \\ \end{array} {\text{ }}} \right.{\text{ }}$$

the function being plotted in Fig. 11. Here *D*_*max*_ denotes the maximal expected depth of pores, 255 − *ε* is the lowest brightness level below of which the pixels are qualified as belonging to pores;

16 $$q = \frac{{D{}_{\hbox{max} }}}{255 - \varepsilon }$$

is a scaling coefficient.

**Fig. 12:**
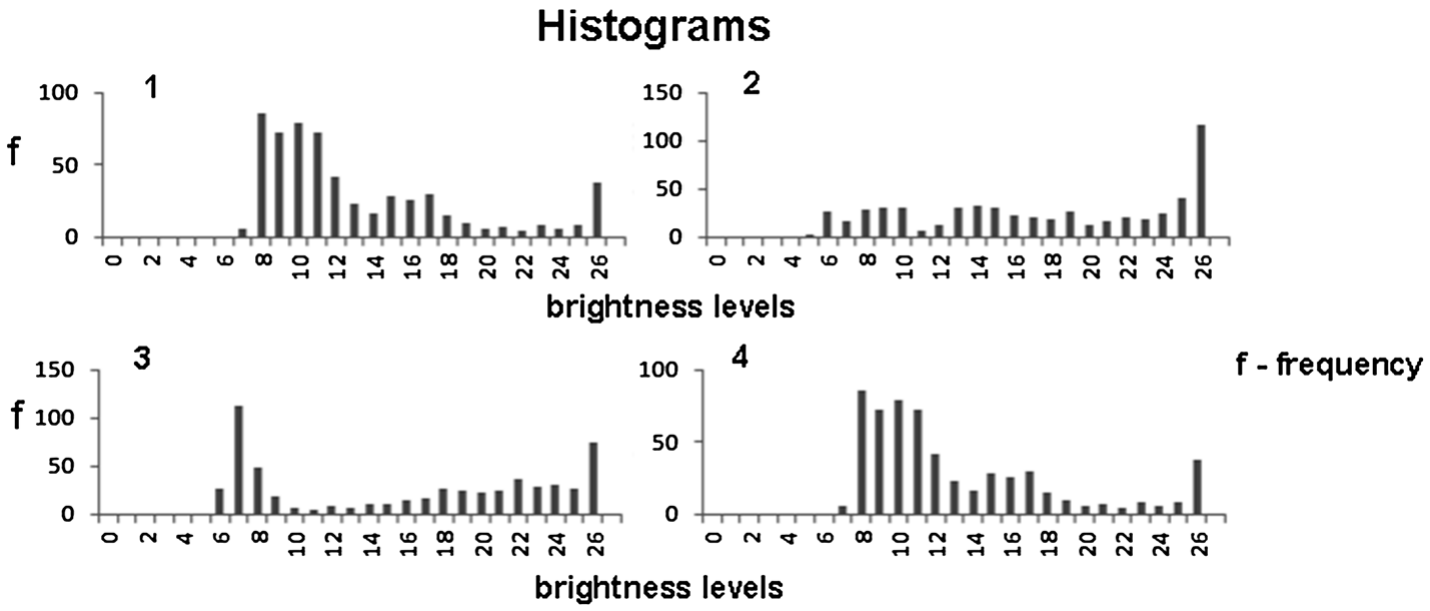
Histograms of brightness levels recorded along the lines 1–4 in Fig. 10a

The constants *D*_*max*_ and *ε* should be established on the basis of image analysis. Figure 12 shows the histograms of brightness levels *β* recorded along the lines 1–4 in the image in Fig. 10a.

The brightness scale of 0–255 units has been divided into sub-intervals by 10 units enumerated from 0 to 26. Each bar in the histogram represents the number of pixels whose brightness levels belong to the given sub-interval.

In all histograms domination of the 26-th bars over the several preceding bars is remarkable. This means that in the given case *ε *= 10 in the formulae (15, 16) can be used.

At the next step, the profiles shown in Fig. 10b should be taken into consideration. On the level 255 − *ε* the lengths *D*_*α*_ of sub-intervals crossing the areas where 0 ≤ *β *<255 − *ε* should be recorded.

The value:

17 $$D_{max} = max\{ D_{a} \}$$

then can be used in the formulae (15, 16). Finally, if *ζ*_*ν*_ *ν* = 1,2,…, *N*, denote the calculated depths of pores and *N* is a total number of pixels in the analyzed image then the value *V*_Σ_ in formula (7) will be given as a sum:

18 $$V_{\sum } = \sum\limits_{\nu = 1}^{N} {\zeta_{v} }$$

because each pixel in the analyzed image represents a cuboid of 1 × 1 × *ζ*_*ν*_ [*pix*] size. For similar reason, it is

19 $$V \, = \, N \cdot 2 5 5,$$

which can be inserted into the formula (7).

## Conclusions

Artificial porous membranes have a wide spectrum of applications in various areas of medicine. Porosity of membranes directly influences their utility as filters separating some types of particles from a fluid matter or as scaffolds for cultivation of biological tissues. However, the notion of porosity contains several properties directly connected with morphological microstructure of pores. The size, density and shape of pores can be mentioned as the basic porosity parameters. Evaluation of the parameters is thus an auxiliary but still important problem to be solved in order to improve the quality of membranes and to increase their utility. However, the problem is not trivial because of irregularity of the shapes of pores and lack of access to some characterizing them data. In this paper some concepts concerning the methods of artificial membranes’ porosity evaluation based on computer-aided analysis of microscope images of their cross-sections are presented. The proposed methods are the result of a long-term cooperation between two working groups in the Nalecz Institute of Biocybernetics and Biomedical Engineering PAS: first (headed by Ph.D. Przytulska), specialized in computer-aided image processing methods and second (headed by prof., D.Sc. Chwojnowski) specialized in artificial membranes design and applications. The methods are based on combined morphological and statistical approach and on some assumptions concerning the statistical isotropy of the materials under investigation. Unlike other methods of 3D pores analysis (e.g. the Focused Ion Beam or Serial Block Face methods) assuming easy access to deep sections of the specimens of examined materials, the proposed methods can be recommended in case of pores analysis based on single SEM images of 2D sections of the materials. It is shown that all the parameters needed to practically use the proposed methods can be obtained by relatively simple numerical analysis of images: measuring distances, areas, brightness profiles, calculation of histograms and their parameters, etc. The necessary procedures are available in various statistical or image processing libraries of programs [23, 24]. However, in order to make our research more effective an original specialized software MeMoExplorer™ has been designed and it is still under development [27].
